# Covert Intention to Answer “Yes” or “No” Can Be Decoded from Single-Trial Electroencephalograms (EEGs)

**DOI:** 10.1155/2019/4259369

**Published:** 2019-07-10

**Authors:** Jeong Woo Choi, Kyung Hwan Kim

**Affiliations:** ^1^Department of Biomedical Engineering, Yonsei University, Wonju 26493, Republic of Korea; ^2^Department of Neurosurgery, University of California, Los Angeles, CA 90095, USA

## Abstract

Interpersonal communication is based on questions and answers, and the most useful and simplest case is the binary “yes or no” question and answer. The purpose of this study is to show that it is possible to decode intentions on “yes” or “no” answers from multichannel single-trial electroencephalograms, which were recorded while covertly answering to self-referential questions with either “yes” or “no.” The intention decoding algorithm consists of a common spatial pattern and support vector machine, which are employed for the feature extraction and pattern classification, respectively, after dividing the overall time-frequency range into subwindows of 200 ms × 2 Hz. The decoding accuracy using the information within each subwindow was investigated to find useful temporal and spectral ranges and found to be the highest for 800–1200 ms in the alpha band or 200–400 ms in the theta band. When the features from multiple subwindows were utilized together, the accuracy was significantly increased up to ∼86%. The most useful features for the “yes/no” discrimination was found to be focused in the right frontal region in the theta band and right centroparietal region in the alpha band, which may reflect the violation of autobiographic facts and higher cognitive load for “no” compared to “yes.” Our task requires the subjects to answer self-referential questions just as in interpersonal conversation without any self-regulation of the brain signals or high cognitive efforts, and the “yes” and “no” answers are decoded directly from the brain activities. This implies that the “mind reading” in a true sense is feasible. Beyond its contribution in fundamental understanding of the neural mechanism of human intention, the decoding of “yes” or “no” from brain activities may eventually lead to a natural brain-computer interface.

## 1. Introduction

The most fundamental linguistic communication consists of questions and answers, and the simplest one is the binary “yes or no” question and answer. This enables fundamental interpersonal communications (e.g., “Is your name John?” “Yes” or “Do you want to drink water?” “No”). So, by decoding the intentions to answer either “yes” or “no” from brain activities, a natural interpersonal communication tool, which does not require any operant training or heavy cognitive efforts, may be developed. As the first step toward this, here we tried to demonstrate that it is possible to decode the intentions to answer “yes” or “no” in response to self-referential questions from noninvasive electroencephalograms (EEGs) on single-trial basis. This was motivated by our recent studies which showed that the intentions to answer “yes” or “no” to self-referential questions is represented significantly differently in event-related EEGs, particularly in alpha-band activities [1, 2].

Direct decoding of “yes” and “no” intentions may eventually lead to advancement of the brain-computer interface (BCI), which is a technological means to deliver user's intention to the external world (device or other people) without behavioral outputs, by direct interpretation of brain activities. The most important target of the BCI is the patients with severe motor impairment, who are unable to communicate with others including those in the completely locked-in state (CLIS) due to amyotrophic lateral sclerosis, spinal cord injury, and brainstem stroke [3–6]. One of the most crucial technologies to enable the BCI is to read or “decode” the users' intention from their brain activities. Two major approaches have been pursued for the intention decoding. The first is based on voluntary self-regulation of specific brain signals such as slow cortical potential [7] and sensorimotor rhythms [8]. This requires extensive operant training using feedback and reward. Unfortunately, many people are unable to regulate the brain activities as required, which is known as “BCI illiteracy” [9, 10]. The other approach utilizes evoked brain activities such as P300 event-related potential (ERP) [11, 12] and steady-state evoked potential [13, 14]. The operant training is not required, but sustained attention is needed to induce discriminable brain response increases, resulting in significant cognitive workload.

Both approaches may not be so successful for the patients with CLIS [15]. It is speculated that the failure is due to the extinction of goal-directed cognition and thought in the CLIS patients [15]. An alternative approach for the mind reading is crucial, which does not require volitional and highly cognitive efforts. Birbaumer and colleagues suggested an approach based on classical conditioning [16–18]. They tried to associate language stimuli with unpleasant and painful sensory stimuli so that cortical responses to these nonlanguage stimuli are conditioned according to the language stimuli. This is remarkable considering that language is the most natural means of communication.

The specific aim of this study is to show that it is feasible to decode “yes” and “no” answers in mind from single-trial EEGs. We demonstrated that mind reading in a true sense, which is based on the prediction of the intentions to answer the questions from brain activities, is achievable. For the intention decoding, the discriminative characteristics of EEGs that we found in our previous study were utilized to find the time-frequency features for “yes/no” decoding. The decoding algorithm was developed based on the same data used in our previous study [2].

## 2. Materials and Methods

### 2.1. Subjects

23 subjects with no record of neurological or psychiatric illness participated in the experiment (age: 23.13 ± 2.97 years, 12 males). All the subjects were undergraduate students of Yonsei University and right-handed native Korean speakers. Written informed consent was obtained from each subject before the experiment. The experimental procedure was approved by the Yonsei University Wonju Institutional Review Board (IRB). All experiments were performed in accordance with the guidelines and regulations of the IRB.

### 2.2. Experimental Task

Before the experiment, all subjects completed a written questionnaire on their autobiographical facts (e.g., job, name, age, and date of birth). We generated two opposite types of questions from a single autobiographical fact; one question should be answered “yes,” and the other (i.e., autobiographical fact violation (AFV)) should be answered “no.” These two questions were almost the same except one critical word (*italicized* word in the example below), which determined whether the question agreed with the subject's identity or not. For example, if the subject's job was a student, the two questions were as follows:

  Type (a), to be answered “yes”: Is your job a *student*?
  Type (b), to be answered “no”: Is your job a *teacher*?

In total, 40 type (a) questions and 40 type (b) questions were generated based on the questionnaire for each subject. Each question was composed of 2 or 3 Korean words, and the average number of characters (Korean “Hangul”) in each critical word was 3.18 ± 1.02. Each character had 3.3 cm width and 4.27 cm height.

All questions were presented visually through commercial software (PRESENTATION; Neurobehavioral systems, Berkeley, CA). After explaining the detailed procedure of the experimental task, we requested the subjects to watch each word presented on a 17 inch computer screen carefully so that they can make immediate response as soon as possible to the critical words. The distance between the subject's eyes and the monitor was set to ∼0.75 m. Each word in a question was presented sequentially one by one on the center of the monitor, as described below.

**Figure 1:**
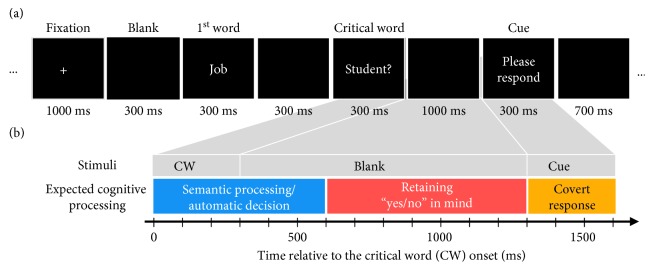
Experimental task: (a) presentation of stimuli; (b) expected temporal sequence of cognitive processing.

Figure 1(a) illustrates the experimental procedure. A cross mark (“+”) for the fixation appeared for 1000 ms and a black screen followed for 300 ms. And then, each word in a question was presented sequentially for 300 ms, with a black screen for 300 ms between the words. The last word in the question is referred to the critical word (CW), which was presented for 300 ms along with a question mark. Although this question mark may naturally induce decision of “yes” or “no” and thus evoke answer automatically, we instructed the subjects not to make any response neither covertly nor overtly but to retain the answer in mind during the 1 s blank period. This would enable us to explore the cortical activity during retaining the information on “yes” or “no” in working memory (WM). Finally, when “Please respond” (in Korean) was presented for 300 ms, the subjects were requested to respond covertly in mind with either “yes” or “no” without any behavioral responses.

Figure 1(b) illustrates expected temporal sequence of cognitive processing following the CW onset until the “Please respond” cue appeared, which was based on our previous studies on cortical information processing of intention [1, 2], which showed that the brain activities differed between “yes” and “no” answers at both early (0∼600 ms) and late periods (600∼1300 ms) relative to the CW onset. We found that the early period was associated with semantic processing and automatic decision to answer [1] (denoted by a red box in Figure 1(b)), while the late period was involved in the retention of the answer in memory (denoted by a blue box in Figure 1(b)) until the “Respond cue” appeared (denoted by a yellow box in Figure 1(b)) [2]. Thus, the temporal period of interest for decoding the intentions to answer “yes” or “no” was the late period, corresponding to retain the intention in mind (600∼1300 ms).

Each subject performed two blocks of tasks. Each block included all questions generated based on the questionnaire (i.e., 40 type (a) and 40 type (b) questions), and 10 of 40 questions for each question type were randomly selected and presented once again. Consequently, each block included 50 type (a) and 50 type (b) questions in total. The average duration of each single trial (i.e., one question and answer) was 4380 ± 274.95 ms. The total time for performing the tasks was approximately 20 minutes including at least 5 minutes of rest between blocks.

### 2.3. Electroencephalogram (EEG) Recording and Data Analysis

Sixty channel EEGs were recorded based on the 10–10 system using an EEG amplifier (Brain Products GmbH, Munich, Germany) with an Ag/AgCl electrode cap (EASYCAP, FMS, Munich, Germany). The ground and reference electrodes were at AFz and linked mastoids, respectively. The impedances of all electrodes were kept under 10 kΩ. The sampling rate was 500 samples/s. A bandpass filter (0.03–100 Hz) and a notch filter (60 Hz) were applied in order to reduce background noise and powerline interferences.

An open source toolbox EEGLAB was used for the whole procedure of preprocessing [19]. First, single-trial EEGs were segmented during the −500∼1300 ms period relative to the critical word onset. By visual inspection, we removed the single-trial waveforms contaminated severely from nonstereotyped artifacts such as drifts and discontinuity. Then, an independent component analysis (ICA) was employed to the remaining single-trial EEGs in order to correct the ocular and muscular artifacts [20]. The group-averaged percentage of the number of epochs remaining per subject was 98.88 ± 3.08% and 97.96 ± 5.86% for “yes” and “no” questions, respectively.

### 2.4. Yes/No Decoding

**Figure 2:**
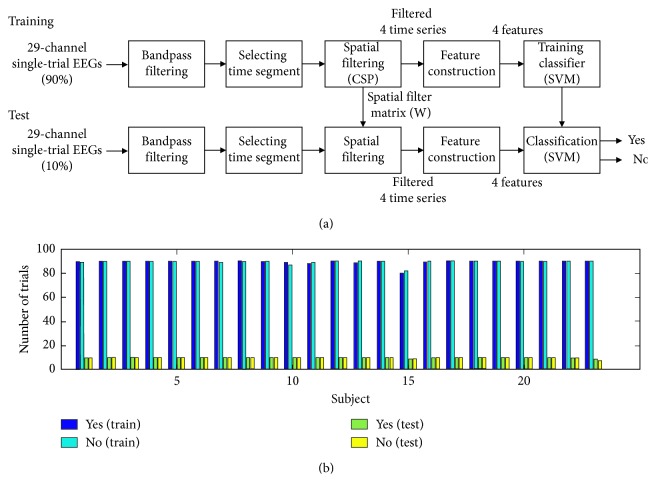
(a) Block diagram of the intention decoding algorithm; (b) the number of trials which was selected as training and testing data within each answer type for each subject.

Figure 2(a) illustrates the structure of “yes/no” intention decoding algorithm using local time-frequency information. First, we selected 29 channels out of 60 (i.e., Fp1, Fpz, Fp2, F7, F3, Fz, F4, F8, FC5, FC1, FC2, FC6, T7, C3, Cz, C4, T8, CP5, CP1, CP2, CP6, P7, P3, Pz, P4, P8, O1, Oz, and O2), following the standard 10–20 system. This was based on a recent simulation study which showed that the decoding accuracy with the common spatial pattern (CSP) spatial filtering was optimized when ∼30 channels were used and decreased for more channels [21]. The overall time-frequency range (0–1200 ms, 4–50 Hz) was divided into subwindows of 200 ms × 2 Hz. The intention decoding within each of the local time-frequency subwindows was performed as follows. Single-trial EEGs were bandpass filtered in the frequency range of the subwindow using a linear-phase finite impulse response filter (the number of the filter order: 512, bandwidth: 2 Hz). The multichannel bandpass-filtered signals within the temporal period of the subwindow were subsequently projected to the lower dimension (four dimensions) by the CSP algorithm [22]. The four time series obtained from the CSP spatial filter were used to construct a four-dimensional feature vector, which was passed to a support vector machine (SVM) classifier. The final output of the classifier was either “yes” or “no,” a decision of answer for each single trial.

The performance of the trained classifier was validated by 10-fold cross-validation as follows: First, for each class, we randomly split all the trials into 10-folds with the same number of trials (i.e., ∼10 trials per fold for each class). Then, we randomly selected one fold (*k* ^th^, where *k* = 1, 2,…, 10) as a test data (10%) and trained the classifier using the rest of data (i.e., 9 folds excepting the *k* ^th^ fold, 90%). In order to keep a balance between the numbers of “yes” and “no” trials, the training/testing data were selected within each class (i.e., “yes” or “no”), as shown in Figure 2(b). The ground truth for each single trial was determined whether the question in the single trial was including AFV or not. The decoding accuracy for each subject was estimated by averaging the ratio of correct classification from 10 repetitions (i.e., *k* = 1, 2,…, 10) of this procedure.

Additionally, we also made effective use all the features obtained from multiple time-frequency subwindows, in order to investigate whether more accurate decoding is possible by combining useful features each of which was localized in the time-frequency domain. The time-frequency subwindows were selected if the decoding accuracies for a specific subwindow were higher than a predetermined threshold (2 × standard deviation above the mean among all time-frequency subwindows). And then, the classifier was trained and tested as described above, with input feature vectors obtained by combining all the selected subwindows.

### 2.5. Event-Related Spectral Perturbation (ERSP) Analysis

The time-frequency activation patterns, i.e., ERSPs, were investigated to reveal statistical differences between “yes” and “no” to find the time and frequency ranges of interests for effective classification. A` continuous wavelet transform (CWT) based on a complex Morlet wavelet was used for the ERSP analysis [23]. The number of cycles for the CWT linearly increased according to the frequency from 4 to 13.5, at the lowest (1 Hz) and the highest frequencies (100 Hz), respectively [19]. This method provides better frequency resolution at high frequencies, and it is better matched to the linear scale that we adopted to visualize the time-frequency map [19]. The induced spectral power was calculated by averaging the ERSP patterns of each single trial [24]. The time-frequency distribution of ERSP patterns was represented as the ratio of the relative change to the power in a baseline interval from −300 to 0 ms prior to stimulus onset, to reduce intersubject variability and to normalize power changes across different frequency bands.

We employed the mass-univariate approach with the cluster-based permutation test for correcting multiple comparisons [25] in order to find the time, frequency, and electrode showing significant differences between “yes” and “no” without *a priori* knowledge. Detailed procedure is as follows:

1. A large number of paired-sample *t*-tests were applied to the data for all time-frequency-electrode bins within the range of 0–1200 ms (time), 5–30 Hz (frequency), and 29 electrodes. The number of bins was 181,714 = 241 × 26 × 29 since there were 241 time samples, 26 frequency points, and 29 electrodes. The electrodes showing high *t* values were selected, and the average power spectral power was calculated over the selected electrodes, as follows. First, from spatial distribution of the *t* values averaged within the frequency band of interest (e.g., theta band: 4–8 Hz; alpha band: 8–13 Hz) during the overall time period (0–1200 ms), the electrodes showing higher *p* values above a predetermined threshold (the upper 10% highest value) were selected. The average power spectral power was calculated over the selected electrodes for the next step.
2. After significant locations were found in step 1, time-frequency bins were screened to be significant among all 6,266 (=241 × 26) bins if *p* values were below a predetermined threshold (*p* < 0.05). A cluster of time-frequency bins was formed if more than two successive bins were selected along either time or frequency axis. Sum of *t* values within the cluster, *t*_mass_, was then calculated and compared with the null distribution of surrogate data to determine statistical significance of the cluster (above the highest 5% of the null distribution). The null distribution of *t*_mass_ was obtained from the largest values of *t*_mass_ for each of 5,000 surrogate data, which were derived by random permutation of “yes” and “no” answers.

### 2.6. Feature Extraction by Common Spatial Pattern (CSP) Filtering

CSP is a mathematical procedure to derive a spatial filter which separates a multichannel signal into additive subcomponents so that the differences of variances are maximized between two classes. That is, the most discriminative features between two classes are obtained by maximizing the variance of the spatially filtered signal of one class while minimizing that of the other class [22]. The CSP algorithm is recognized to be effective for the discrimination of mental states from event-related EEG spectral powers [26]. The results of the CSP can be visualized as a topographic map on the scalp, which facilitates interpretation of functional neuroanatomical meanings [26].

The CSP spatial filter, **W**, can be obtained by simultaneous diagonalization of two covariance matrices of classes 1 and 2 as follows:

(1) $$\begin{array}{c} W^{\text{T}}\Sigma_{1}W=\Lambda_{1},\\ W^{\text{T}}\Sigma_{2}W=\Lambda_{2}, \end{array}$$

where Λ_1_+Λ_2_=**I**. Σ_1_ and Σ_2_ represent the spatial covariance matrices averaged over all single-trial EEGs for each class, and Λ_1_ and Λ_2_ denote the diagonal matrices. The projection vector, *w* (column vectors of **W**), can be obtained from a generalized eigenvalue decomposition as follows:

(2) $$\begin{array}{c} \Sigma_{1}w_{k}=\lambda_{k}\Sigma_{2}w_{k}, \end{array}$$

where **w**_*k*_ (*k* = 1,…, *C*, where *C* is the number of channels) is the generalized eigenvector, and *λ*_1,*k*_=**w**_*k*_^T^Σ_1_ **w**_*k*_ and *λ*_2,*k*_=**w**_*k*_^T^Σ_2_ **w**_*k*_ are defined as the *k* ^th^ diagonal element of **Λ**_1_ and **Λ**_2_, respectively, where *λ*_*k*_=*λ*_1,*k*_/*λ*_2,*k*_. Importantly, *λ*_1,*k*_ and *λ*_2,*k*_ (ranges from 0 to 1) reflect the variance for each class and *λ*_1,*k*_+*λ*_2,*k*_=1. Thus, if *λ*_1,*k*_ is close to 1, *λ*_2,*k*_ should be close to 0. This means that corresponding projection vector, **w**_*k*_, shows high variance in class 1 but low variance in class 2. The difference in variances between these two classes enables discriminating one class from another. The eigenvalues are sorted in the descending order during calculation, meaning that the first projection vector yields the highest variance for class 1 (but the lowest for class 2), whereas the last projection vector yields the highest variance for class 2 (but lowest for class 1). Thus, the first and last projection vectors are the most useful for the discrimination [22].

The spatial filter **W** provides the decomposition of a single-trial multichannel EEG, **E**, as **Z** = **W** ^T^ **E**, where **E** is represented as a matrix with *C* (the number of channels) rows and *T* (the number of time samples) columns. The columns of **W** ^−1^ form the common spatial patterns and can be visualized as topographies on scalp. The variances of the spatially filtered time-series **Z** are calculated as features for the classification as follows:

(3) $$\begin{array}{c} {\text{f}}_{p}=\log\left(\frac{\mathrm{var}\left(Z_{p}\right)}{\sum_{i=1}^{2m}\mathrm{var}\left(Z_{i}\right)}\right),\;\text{where}\;\;p=1,2,\ldots ,2m, \end{array}$$

where *p* is the number of features. *m* was set to 2 which means that the first 2 and last 2 projection vectors were used as features, and thus, the number of features *p* was 4 for all classifications. The log transformation was adopted to approximate the normal distribution of the data.

### 2.7. Pattern Classification Using Support Vector Machine (SVM)

SVM has been recognized to be a practical and robust method for the classification of human brain signals [27, 28]. The SVM is trained to determine an optimal hyperplane by which the distance to the support vectors (closest to the separating boundary) is maximized [29, 30]. In the case of the linear SVM classification, the hyperplane **a** ^T^ **x** + *b* satisfies

(4) $$\begin{array}{c} y_{i}\left(a^{\text{T}}x_{i}+b\right)\geq 1-\xi_{i},\;\text{for}\;\;i=1,\ldots ,N, \end{array}$$

where **x**_*i*_ = {*f*_*p*,*i*_} denotes a feature vector (in which *p* = 1,…, 4) which can be obtained from the CSP algorithm and *y*_*i*_ ∈ {+1, −1} denotes its correct class label. *N* and *ξ*_*i*_ denote the total number of training samples and the deviation from the optimal condition of linear separability, respectively. The pair of hyperplanes that provide the maximum separating margin can be found by minimizing the cost function (1/2)**a** ^T^ **a**+*P*∑_*i*=1_^*N*^ *ξ*_*i*_ subject to the constraints

(5) $$\begin{array}{c} y_{i}\left(a^{\text{T}}x_{i}+b\right)\geq 1-\xi_{i},\\ \xi_{i}\geq 0,\;\text{for}\;\;i=1,\ldots ,N, \end{array}$$

where *P* > 0 represents a regularization penalty parameter of the error term. By transforming this optimization problem into its dual problem, the solution may be determined as **a**=∑_*i*=1_^*N*^ *α*_*i*_ *y*_*i*_ **x**_*i*_ and achieves equality for nonzero values of *α*_*i*_ only. The corresponding data samples are referred to as support vectors, which are crucial to identify the decision boundary. Instead of the basic linear SVM, we used a radial basis function (RBF) kernel which nonlinearly projects the feature vectors onto a higher dimensional space and thus is better suited for nonlinear relationships between features and class labels [29]. The detailed parameters of the SVM including the RBF kernel parameter and regularization penalty were determined by trial-and-error.

## 3. Results

### 3.1. Yes/No Decoding

**Figure 3:**
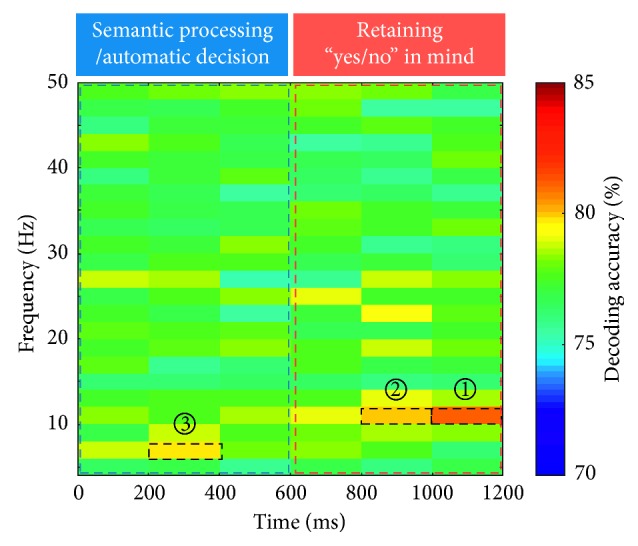
Decoding accuracies of time-frequency subwindows. Color code denotes the decoding accuracy averaged over 23 subjects within each time-frequency subwindow. The best three subwindows are denoted by dashed boxes (①:10–12 Hz, 1000–1200 ms; ②: 10–12 Hz, 800–1000 ms; ③: 6–8 Hz, 200–400 ms).

**Figure 4:**
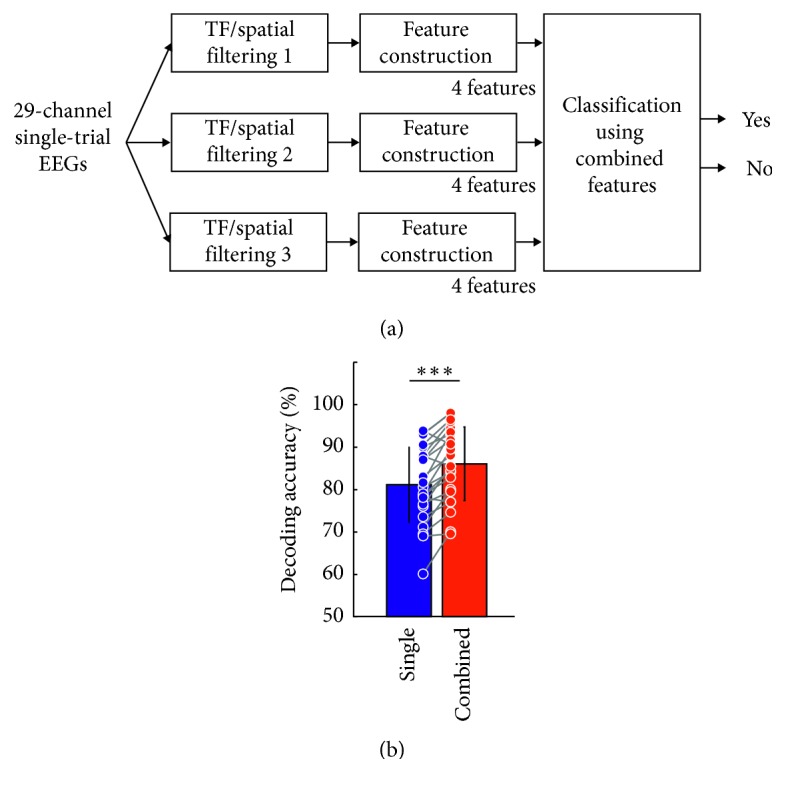
Intention decoding using the features from multiple time-frequency subwindows: (a) intention decoder using the features from multiple time-frequency subwindows; (b) statistical comparison of the decoding accuracies between the cases of using single and multiple time-frequency subwindows (^*∗∗∗*^ *p* < 0.001, by the paired-sample *t*-test; error bar: standard deviation).

Figure 3 shows the time-frequency representation of the “yes/no” decoding accuracy averaged over all subjects for each time-frequency subwindow. The time-frequency map of decoding accuracy was generated by representing the decoding accuracies averaged over all subjects within each time-frequency subwindow, which enables estimation of the decoding accuracies over all time-frequency ranges. We used two criteria to define the most important time-frequency subwindows showing high decoding accuracies. The first was to use the threshold level of a decoding accuracy of 75%, determined by the theoretical 95% confidence limits of the chance level when 10 trials per class are used for testing [31]. Another criterion was that the decoding accuracy should be above the mean + 2 × standard deviation value (79.34% here). The high decoding accuracies above these two threshold levels were obtained for three subwindows in the alpha and theta bands (as denoted by the three boxes in Figure 3) for both early and late periods. The highest and second highest decoding accuracies were found in the upper alpha band (10–12 Hz) at late epoch (box ①: 81.08 ± 8.89% at the 1000–1200 ms, box ②: 79.99 ± 8.99% at the 800–1000 ms). Also, the third highest decoding accuracy was found in the upper theta band (6–8 Hz) at the early period (box ③: 79.76 ± 10.21% at the 200–400 ms). When all 12 features within these three best time-frequency subwindows were used together, the decoding accuracy was drastically enhanced compared to the best subwindow (10–12 Hz, 1000–1200 ms), as shown in Figure 4(a) (single: 81.08 ± 8.89%, combined: 86.03 ± 8.69%, *t*(22) = −5.95, *p* < 0.001, by the paired-sample *t*-test). The individual decoding accuracies are presented in Table 1. The sensitivity and specificity values for each time-frequency subwindow are presented in Supplementary Figure 1.

### 3.2. Spatial Patterns

**Figure 5:**
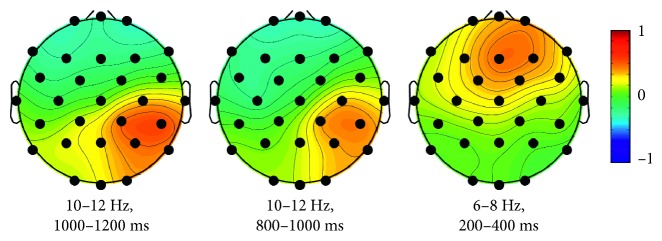
Difference between the most important common spatial patterns for “no” and “yes” answers averaged over all subjects within 3 time-frequency subwindows. The topography was obtained from the difference between the last (“no” answer) and first (“yes” answer) columns of the inverse of the matrix, **W**, for each subject and then averaged over all subjects.

Figure 5 shows the difference between the most important common spatial patterns for “no” and “yes” answers within the three time-frequency subwindows (averaged over all subjects). Each topography was obtained from the difference between the last (“no” answer) and first columns (“yes” answer) of the inverse matrix of the projection matrix, **W**, for each subject (Supplementary Figure 2), which was calculated in each time-frequency subwindow and then averaged over all subjects. The difference between the most important common spatial patterns in the alpha band showed the strongest coefficient at the right centroparietal region at both 1000–1200 ms and 800–1000 ms periods (the leftmost and middle panels in Figure 5, respectively). The difference between the most important common spatial pattern in the theta band at the 200–400 ms period was focused in the right frontal regions (the rightmost panel in Figure 5).

### 3.3. Event-Related Spectral Perturbation (ERSP) Analysis

**Figure 6:**
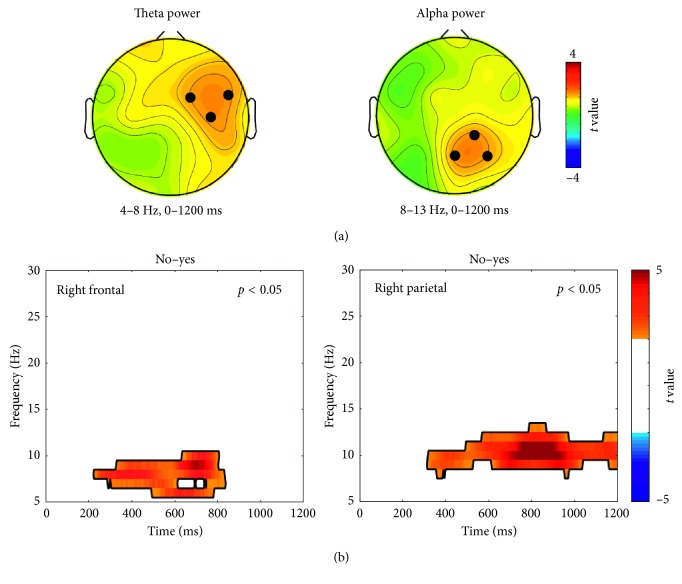
Statistical comparisons in spectral power between “yes” and “no.” (a) Topographical distributions of *t* values (left: theta band (4–8 Hz), 0–1200 ms; right: alpha band (8–13 Hz), 0–1200 ms). Black dots (FC2, FC6, and C4 in the left panel and CP2, Pz, and P4 in the right panel) denote high *t* values above a predetermined threshold, corresponding to the highest 10%. (b) The clusters in the time-frequency domain showing significant differences between “yes” and “no” in right frontal (left panel) and right parietal regions (right panel) (denoted by black contours).

Figure 6(a) shows the topographical distributions of *t* values averaged within the theta band (4–8 Hz) in 0–1200 ms. The 3 electrodes (FC2, FC6, and C4) showing high *t* values above a predetermined threshold (*t* > 1.62, corresponding to the highest 10%) were selected over the right frontal region (denoted by black dots in the left panel in Figure 6(a)). Significant “yes/no” difference was found within a single time-frequency range around 200–800 ms in the upper theta and lower alpha bands (6–10 Hz), which was stronger for “no” compared to “yes” (denoted by a solid contour in the left panel in Figure 6(b)).

In the alpha band, 3 electrodes in right parietal area (CP2, Pz, and P4) with high *t* values were selected as described above (*t* > 1.52, the highest 10%) as denoted by black dots in the right panel in Figure 6(a). The “yes/no” difference in spectral power in this region was significant within a single time-frequency range (300–1200 ms, 9–12 Hz), where the alpha-band power was stronger for “no” compared to “yes” (denoted by a solid contour in the right panel in Figure 6(b)).

## 4. Discussion

We showed that it is possible to decode the intentions to answer “yes” and “no” with high accuracy from single-trial EEGs. The best decoding accuracy averaged over 23 subjects was as high as 86.03% when useful features in multiple time-frequency subwindows were all combined. The decoding accuracy was above 70% for most of the subjects (22 out of 23 subjects), which is considered as a reasonable accuracy for the binary classification [32]. We decoded the “yes” and “no” answers directly from the brain activities representing the two different answers, which implies that the “mind reading” in a true sense is feasible. The experimental paradigm of our study is based on a natural task which required the subjects to answer self-referential questions as in conversation with others, without any self-regulation of the brain signals or high cognitive efforts. No unpleasant stimuli and volition or high cognitive efforts are required since our approach is based on a direct decoding of “yes” and “no” without any self-regulation of the brain signals. Birbaumer's group has suggested a new alternative approach based on classical conditioning to solve the problem of conventional BCI in the CLIS patients [16–18]. For the training, two distinct unconditioned stimuli are presented to the subjects immediately after the simple “yes/no” questions (corresponding to the conditioned stimuli) so that the cortical responses can be conditioned differently for yes and no. The unconditioned stimuli include auditory pink noise and white noise [16, 18] and weak electrical stimulation to the thumb [17]. The main idea of this approach is to modulate the users' brain activities indirectly through the unconditioned stimuli so that “yes” and “no” can be easily discriminated from neural signals responding to the sensory stimuli, rather than to read the users' answers from neural signals. This approach may provide an alternative to the conventional BCI approaches in that volition, or high cognitive efforts are not required. However, it remains unclear how long the conditioned cortical response can be maintained considering the extinction effect of classical conditioning [33]. Moreover, unconditioned stimuli such as auditory noise or electrical stimulation can evoke significant displeasure.

Recently, a more natural approach for the “yes/no” decoding was demonstrated based on functional near-infrared spectroscopy (fNIRS) in the CLIS patients [34]. They achieved “yes/no” decoding accuracy over 70% based on fNIRS signals, which were recorded, while the patients answered “yes” or “no” to personal and open questions in minds repeatedly. Interestingly, for the same experimental protocol, they reported that EEG-based decoding yielded accuracy below the chance level. This study employed a natural question/answer task which does not require high cognitive efforts or volition, just as ours. But due to the slow nature of hemodynamics, the duration of each trial for the decoding was quite long (>10 sec). Here, we showed the possibility of “yes/no” decoding from considerably shorter signal recording, which is more beneficial for a practical BCI communication tool.

We took a systematic approach of finding features of brain activities reflecting “yes/no” answers in minds and then developing the decoding algorithm by utilizing these features. Further studies may be necessary to investigate whether the patients, who would potentially benefit from the BCI, can hold the intentions to answer in minds for a short time and to validate our method on the patients' data.

In this study, the intentions regarding self-referential questions based on the autobiographic facts were investigated. It is important to further try decoding the intentions to answer various types of questions including desire, feeling, and preference. In addition, our questions were presented only in visual stimuli. Neurological patients may have an abnormal visual function such as disability to fix their gaze on specific visual stimuli [35]. Different sensory modality such as auditory stimuli has been tried for the BCI communication tools [34, 36]. It would be beneficial if our approach can be validated with auditory stimuli such as voice, considering that a high decoding accuracy above 80% was obtained even when the brain activities during the period of retaining the decision in minds (10–12 Hz, 1000–1200 ms) used for the decoding. Thus, we expect that it is possible to decode the “yes” and “no” intentions in a similar way, even if other types of questions and/or the auditory stimuli are employed in the further studies. In addition, here, we did not try to optimize the detailed parameters of the SVM, including the RBF kernel parameter and regularization penalty. The use of the best parameters of the SVM, for example, by using the “grid-search” method [37], may be obviously helpful for better results.

We found two time-frequency regions containing useful information for the “yes/no” decoding, in early theta and late alpha bands. The useful features for the “yes/no” decoding in the alpha band were found to be concentrated in the parietal region at 800–1200 ms from the CSP algorithm. Recently, we showed that the alpha rhythms in the right parietal region are differentiated between the intentions to answers either “yes” or “no” in minds, presumably due to the difference in cognitive loads for the WM retention [2]. Several previous studies showed that the higher parietal alpha power reflects increased memory load [38, 39] or attentional demand [40, 41] during WM retention. The higher alpha power is attributed to active inhibitory control to block incoming stimuli during WM retention, for efficient cortical information processing [38, 39, 42, 43]. Our results showed higher parietal alpha power for “no” compared to “yes,” which may imply higher cognitive load during retaining “no” in minds compared to “yes” [2]. The greater increase in alpha-band activity for “no” may reflect the increased WM load during the intention retention. In Korean language, “yes,” is the one-character word, “네,” and “no” is three-character word, “아니오.” It is plausible that the higher WM load is required to represent intention to respond “no” than “yes” due to the length of the Korean words, resulting in the higher alpha rhythm. This assumption is supported by an ERP study which reported that greater alpha-band power was induced for retaining longer word [44].

It can also be interpreted that the significantly higher alpha-band activity in the centroparietal region for “no” is due to the higher attentional demand [40, 45], and this contributed to the high decoding accuracy. This is also in agreement with a recent study [46], which reported that a higher alpha rhythm was identified in the right parietal cortex for a higher internal attention condition during a divergent thinking task. Our result of greater alpha power for “no” than for “yes” may imply a stronger inhibition of the outer stimuli by the bottom-up attention network for “no,” induced by higher internal attentional demand. This is supported by psychophysical which showed that saying “no” requires more effortful reconsideration after comprehending a sentence and a longer response time for saying “no” than “yes” [47, 48].

The theta-band activity in the frontal region in 200–500 ms was another major feature for “yes/no” decoding. The theta ERS showed topography focused on midline frontal and lateral temporal regions. The difference between “yes” and “no” was also most prominent in these regions. Hald et al. reported that temporal and frontal theta-band activity in 300–800 ms was significantly higher in semantically incongruent compared to congruent sentences [49]. This is commensurate with our result in that “no” stimuli are incongruent with autobiographic facts. The increase of theta-band activity for semantic incongruence was interpreted to reflect the general error detection mechanism, which is associated with error-related negativity (ERN) [50]. Interestingly, Luu and Tucker showed that frequency domain analysis of the ERN yields theta-band activity in the midfrontal region [50]. A related study reported higher theta oscillation for syntactic violation as well [51]. We observed that frontal theta power in 200–500 ms contributed to high decoding accuracy. Considering the location and frequency band, our result on the usefulness of frontal theta power in 200–500 ms can be interpreted as another evidence, suggesting that error-related frontal theta oscillation is a general phenomenon underlying processing of incoming stimuli containing violation with internal information.

## Figures and Tables

**Table 1:** Decoding accuracies for individual subjects.

Subject	TF subwindow ①	TF subwindow ②	TF subwindow ③	Combined features
1	86.89 ± 6.52 (78.95, 100)	88.95 ± 4.35 (80.00, 95.00)	87.37 ± 9.47 (70.00, 100)	94.50 ± 3.50 (90.00, 100)
2	71.00 ± 6.63 (55.00, 80.00)	74.50 ± 10.11 (55.00, 90.00)	78.00 ± 9.80 (65.00, 95.00)	80.00 ± 10.49 (60.00, 95.00)
3	76.00 ± 10.20 (60.00, 90.00)	79.00 ± 6.63 (65.00, 90.00)	82.00 ± 7.14 (65.00, 90.00)	85.50 ± 7.89 (65.00, 95.00)
4	80.50 ± 8.79 (65.00, 95.00)	75.50 ± 7.23 (65.00, 90.00)	71.00 ± 8.00 (60.00, 85.00)	84.00 ± 3.74 (80.00, 90.00)
5	88.00 ± 3.32 (85.00, 95.00)	89.00 ± 10.44 (65.00, 100)	92.00 ± 7.48 (75.00, 100)	96.00 ± 4.90 (85.00, 100)
6	90.00 ± 7.07 (75.00, 100)	90.50 ± 4.15 (85.00, 95.00)	92.00 ± 5.57 (85.00, 100)	93.00 ± 4.00 (85.00, 100)
7	78.47 ± 13.21 (45.00, 94.74)	77.45 ± 10.00 (65.00, 100)	77.42 ± 9.75 (60.00, 90.00)	84.45 ± 8.17 (65.00, 95.00)
8	83.00 ± 5.57 (75.00, 95.00)	75.50 ± 7.57 (65.00, 85.00)	80.50 ± 7.57 (65.00, 90.00)	88.00 ± 8.72 (75.00, 100)
9	73.50 ± 9.76 (55.00, 85.00)	70.00 ± 8.37 (60.00, 85.00)	68.00 ± 6.40 (55.00, 80.00)	77.00 ± 8.12 (65.00, 90.00)
10	78.64 ± 10.95 (60.00, 94.74)	68.54 ± 8.60 (60.00, 89.47)	76.09 ± 12.77 (50.00, 94.74)	82.73 ± 6.25 (70.00, 90.00)
11	76.54 ± 7.58 (63.16, 85.00)	76.34 ± 9.54 (60.00, 89.47)	67.59 ± 6.00 (60.00, 75.00)	79.54 ± 6.25 (66.67, 90.00)
12	92.95 ± 3.37 (89.47, 100)	94.00 ± 7.35 (75.00, 100)	95.97 ± 3.75 (90.00, 100)	97.97 ± 2.48 (94.74, 100)
13	87.89 ± 6.54 (78.95, 100)	82.84 ± 9.76 (65.00, 95.00)	74.42 ± 12.27 (50.00, 90.00)	85.45 ± 8.17 (70.00, 100)
14	60.00 ± 7.75 (45.00, 75.00)	65.00 ± 10.25 (45.00, 75.00)	69.50 ± 14.04 (50.00, 90.00)	70.00 ± 10.49 (50.00, 85.00)
15	69.58 ± 14.98 (44.44, 88.89)	79.53 ± 10.70 (55.56, 94.44)	58.96 ± 13.75 (33.33, 77.78)	74.55 ± 10.76 (57.89, 94.44)
16	68.95 ± 8.49 (60.00, 90.00)	64.51 ± 6.80 (50.00, 75.00)	67.45 ± 8.21 (55.00, 80.00)	69.48 ± 7.00 (60.00, 85.00)
17	79.00 ± 9.43 (65.00, 90.00)	77.00 ± 7.14 (65.00, 85.00)	83.00 ± 9.00 (70.00, 95.00)	89.50 ± 7.89 (80.00, 100)
18	81.50 ± 8.67 (65.00, 100)	80.00 ± 9.22 (60.00, 90.00)	88.00 ± 7.14 (70.00, 95.00)	93.50 ± 5.50 (80.00, 100)
19	93.00 ± 5.10 (85.00, 100)	97.00 ± 4.00 (90.00, 100)	94.50 ± 2.69 (90.00, 100)	98.00 ± 2.45 (95.00, 100)
20	90.50 ± 4.72 (85.00, 100)	89.50 ± 6.10 (80.00, 100)	88.50 ± 6.73 (75.00, 100)	96.50 ± 4.50 (85.00, 100)
21	87.00 ± 7.48 (70.00, 95.00)	83.00 ± 9.27 (65.00, 100)	83.50 ± 5.50 (75.00, 95.00)	91.50 ± 5.94 (80.00, 100)
22	78.00 ± 8.72 (60.00, 90.00)	73.50 ± 8.08 (55.00, 85.00)	70.00 ± 8.66 (55.00, 85.00)	77.00 ± 4.00 (70.00, 85.00)
23	93.82 ± 5.51 (87.50, 100)	88.62 ± 8.34 (66.67, 100)	88.77 ± 6.86 (75.00, 100)	90.61 ± 9.01 (73.33, 100)
Ave	81.08 ± 8.89 (60.00, 93.82)	79.99 ± 8.99 (64.51, 97.00)	79.76 ± 10.21 (67.45, 95.97)	86.03 ± 8.69 (69.48, 98.00)

Value: mean ± standard deviation (SD) of decoding accuracy (%). The range of decoding accuracies was in parenthesis. Abbreviation: TF, time frequency; Ave, average values over all subjects.

## Data Availability

The data used to support the findings of this study have not been made available because some participants of this study did not agree to distribute their physiological signals.

## Supplementary Materials

Supplementary Materials The supplementary figures demonstrate the sensitivity and specificity of “yes/no” decoding for each time-frequency subwindow (Supplementary Figure 1) and the difference between the most important spatial patterns discriminating “yes” and “no” for the individual subjects at the three time-frequency subwindows (Supplementary Figure 2).
