# Supplementary material for: Covert Intention to Answer “Yes” or “No” Can Be Decoded from Single-Trial Electroencephalograms (EEGs)
Source: Comput Intell Neurosci. 2019 Jul 10;2019:4259369. doi: 10.1155/2019/4259369 (PMC6652077; doi:10.1155/2019/4259369)
Supplement: Supplementary Materials — The supplementary figures demonstrate the sensitivity and specificity of “yes/no” decoding for each time-frequency subwindow (Supplementary Figure 1) and the difference between the most important spatial patterns discriminating “yes” and “no” for the individual subjects at the three time-frequency subwindows (Supplementary Figure 2). [file 4259369.f1.docx]

**Supplementary Materials**


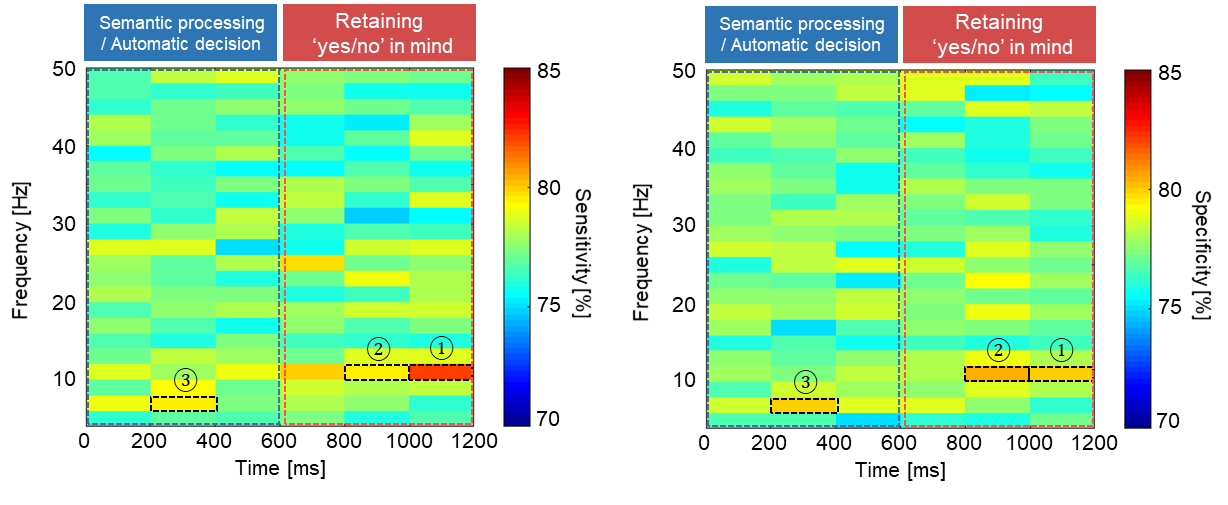


**Supplementary Figure 1**: (a) Sensitivity and (b) specificity of time-frequency sub-windows. Color code denotes the values averaged over 23 subjects within each time-frequency sub-window. Dashed boxes represents the three sub-windows showing the best decoding accuracies in Figure 3 (①:10-12 Hz, 1000-1200 ms, ②: 10-12 Hz, 800-1000 ms, and ③: 6-8 Hz, 200-400 ms).


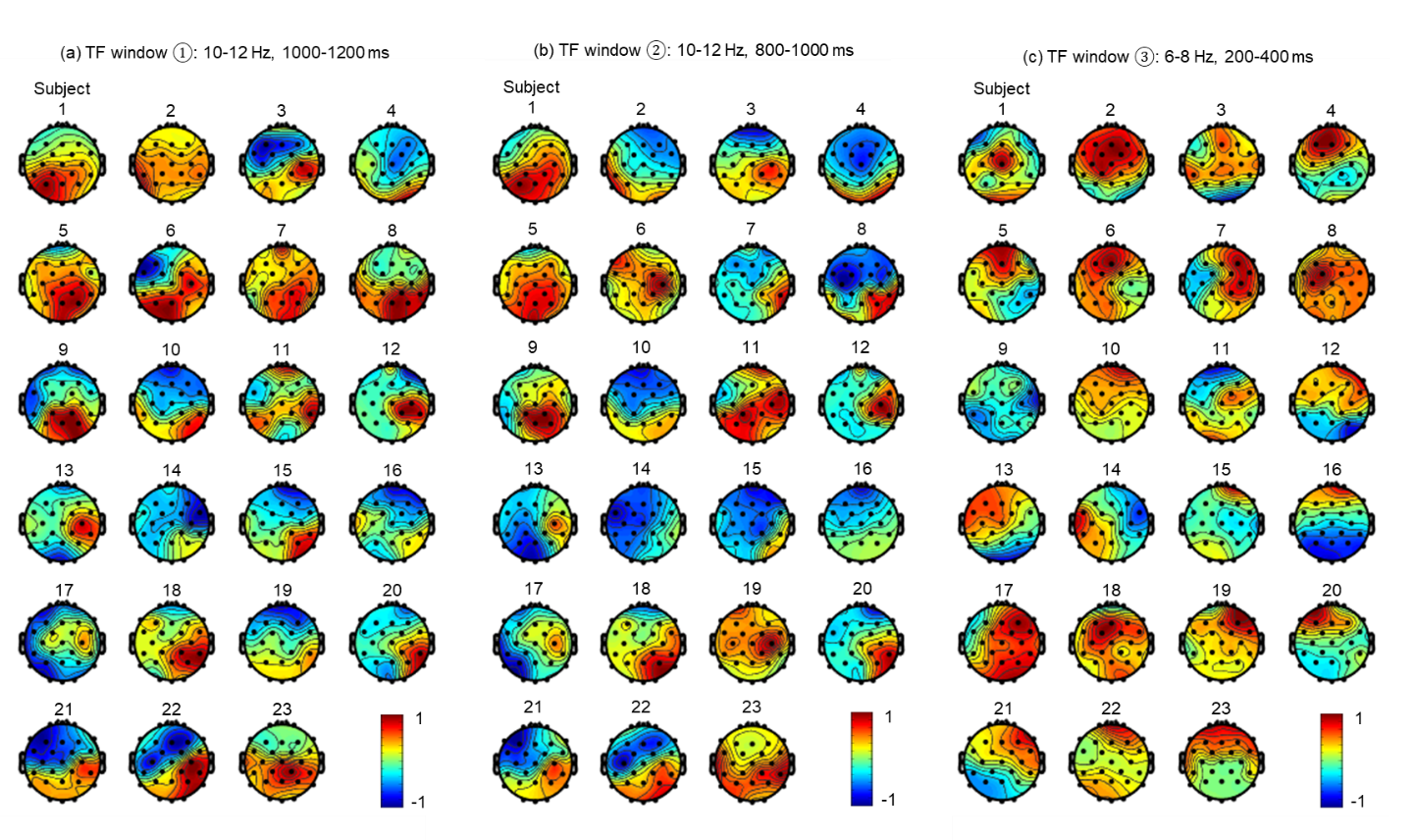


**Supplementary Figure 2**: The difference between the most important spatial patterns between ‘no’ and ‘yes’ answers for individual subjects at (a) time-frequency sub-window ①, (b) time-frequency sub-window ②, and (c) time-frequency sub-window ③ as illustrated in Figure 3. Figure 5 was obtained by averaging these individual topographical maps within each time-frequency sub-window.
